# The Outbreak of Unexplained Acute Hepatitis in Children: The Role of Viral Infections in View of the COVID-19 Pandemic

**DOI:** 10.3390/v16050808

**Published:** 2024-05-20

**Authors:** Eyal Shteyer, Orna Mor, Orith Waisbourd-Zinman, Yael Mozer-Glazberg, Ronen Arnon, Lior Hecht Sagie, Michal Mandelboim, Oran Erster, Merav Weil, Sara Dovrat, Lital Goldberg, Yael Gozlan

**Affiliations:** 1 The Juliet Keidan Institute of Pediatric Gastroenterology Institute, Shaare Zedek Medical Center, Jerusalem 9103102, Israel; 2 Centeral Virology Laboratory, Israeli Ministry of Health, Sheba Medical Center, Ramat-Gan 52620000, Israel; 3 Faculty of Medicine, Tel Aviv University, Tel-Aviv 69978, Israel; 4 Gastroenterology, Nutrition and Liver Diseases, Schneider Children’s Medical Center, Petach-Tiqva 4920235, Israel; 5 Pediatric Gastroenterology Unit, Rambam Medical Center, Haifa 3109601, Israel; 6 Division of Epidemiology, Israeli Ministry of Health, Jerusalem 9101002, Israel

**Keywords:** acute hepatitis of unknown origin, COVID-19, human herpes virus 6

## Abstract

Background and Aims: An increase in the number of cases of acute hepatitis of unknown origin (HUO) in children was observed in 2021. Adenovirus and adeno-associated virus 2 (AAV2) infections have been suggested as possible triggers. However, the potential etiology is still unclear. We aimed to characterize a cohort of children with HUO in Israel in view of the COVID-19 pandemic. Method: Demographics, clinical data, and laboratory results on the children compatible with the CDC criteria for HUO were collected by the established registry of the Ministry of Health. Available specimens were sent to the Central Virology Laboratory. Results: A total of 39 children were included in the registry. A total of 20 were enrolled prospectively, in which human herpes virus 6 (HHV6) infection or reactivation was identified in 11/19, adenovirus was found in 4/19 of the cases, and AAV2 was detected in 2/16. Past COVID-19 exposure was recorded for 24/39 of the children. A total of 10 children underwent liver biopsy, and 8 were successfully treated with steroids and 2 underwent liver transplantation. Conclusions: The COVID-19 pandemic and the related containment measures combined with reactivation or active infection with other viruses could have been a trigger for the HUO outbreak. In our cohort, HHV6 was the most abundant finding.

## 1. Introduction

An initial report from the United Kingdom in early April 2022 of an increased number of children with acute hepatitis of unknown origin (HUO) gained attention worldwide and led to reports of more than 1000 children with HUO two months later. An interim review of the published cases up to June 2022 [1] showed that 45 cases (5%) of the 920 probable cases required liver transplantation and 18 cases (2%) died. An international online survey affirmed the impression that there was indeed an increase in acute hepatitis in 2022 compared to the previous 5 years. The first report of a plausible cause came from Alabama, USA, suggesting adenovirus as the cause for this outbreak [2]. Accordingly, it was postulated that the preceding COVID-19 infection or the changed immunity of children due to lockdowns during the pandemic may have had a role in the pathogenesis of HUO [3].

Further global investigations searching for the agent causing HOU showed that adeno-associated virus 2 (AAV2) was present in many of the children tested and most had coinfection with additional viruses, of which, adenovirus was the most prominent [4,5,6]. However, as adenovirus is abundant in children and rarely causes severe liver disease, these findings may be incidental. Nevertheless, AAV2 was a significantly less abundant finding in the control groups [4,5,6], and the coinfections with additional viruses suggested that the severe hepatitis is immune-mediated. Human herpes virus 6 (HHV6), which infects 90% of infants before the second year of life [7], was another pathogen reported in some of these HUO cases. Subsequent to primary infection, the virus is usually maintained in a latent form, and different triggers result in the reactivation of this virus [7,8]. Their analysis suggests that coinfections of childhood pathogens may predispose children to developing this novel severe hepatitis. Altered susceptibility and response to such pathogens may be a consequence of immunological naivety following pandemic restrictions. The need for further investigations on the etiology of different patient demographics and geographical areas is highlighted [9].

The COVID-19 pandemic’s impact on the transmission of various pathogens has been observed worldwide [10]. Israel’s distinctive response to the pandemic may have influenced the spread of viral infections in the country. Israel was among the first nations to commence COVID-19 vaccinations, emphasizing the importance of prompt action in outbreak management. Early in the pandemic, the Ministry of Health (MoH) in Israel implemented rigorous containment measures, including multiple lockdowns, stringent travel restrictions, widespread testing, self-isolation protocols, and mandatory mask-wearing [11]. Consequently, despite the early arrival of COVID-19 in February 2020, Israel has been widely commended for its effective pandemic response.

Our aim in the current study was to describe an Israeli cohort of children with HUO and to assess viral pathogens previously related to HUO against the background of the COVID-19 pandemic.

## 2. Methods

### 2.1. Inclusion Criteria

Subsequent to the WHO/CDC announcement on HUO, a national registry was established by the Israeli Ministry of Health. The first patients were already described in February 2021 [12]. This report was compatible with the pediatric hepatology community in Israel, and therefore, retrospective data were collected starting from April 2021 and prospective data starting from April 2022. Inclusion criteria were the lack of a confirmed etiology; liver enzyme levels (aspartate aminotransferase (AST) or alanine aminotransferase (ALT) > 500 U/L; age < 18 years; and onset on or after April 1, 2021. Demographic and clinical data and laboratory results were collected and, when available, blood, serum nasopharyngeal, and stool samples were sent to the HIV and viral hepatitis national reference center in the central virology laboratory (CVL).

### 2.2. Ethical Approval

The study was approved by the local Institutional Review Board (IRB, Helsinki Committee) of Shaare Zedek Medical Center, Jerusalem, Israel, (No. 0154-22-SZMC). This study was granted a waiver of informed consent.

### 2.3. Laboratory Tests

COVID-19 status was determined according to the Ministry of Health registry. A positive COVID-19 PCR result, positive antigen result, or positive serology result was determined as exposure. Whole-blood, sera, stool, and nasopharyngeal samples available from 20 patients (Table S1) were analyzed in the CVL. Nucleic acids from blood, sera, and nasopharyngeal samples were extracted using the MagNA Pure 96 System (Roche, Vienna, Austria) or MagDEA^®^ Dx SV (Precision System Science Co., Ltd., Matsudo, Japan) and from stool using NUCLISENS^®^ EASYMAG^®^ (bioMérieux, Hampshire, UK), according to the manufacturer’s instructions. Qualitative real-time PCR (RT-PCR) was used for the detection of adenovirus, herpes virus 1 and 2, human herpes virus 6 (HHV6), cytomegalovirus (CMV), and Epstein–Barr virus (EBV) in whole blood and in plasma and adenovirus, enterovirus, norovirus, and rotavirus also in stool using previously described protocols [13,14,15]. AAV2 was tested in all available nucleic acid extracts by qualitative RT-PCR [16]. Respiratory viruses (adenovirus, influenza A virus, influenza B virus, metapneumovirus, parainfluenza virus, respiratory syncytial virus, human rhinovirus) were detected in nasopharyngeal swabs using the Allplex™ RV7 Essential Assay (Seegene Inc., Seoul, Republic of Korea).

HHV6 serology was performed using indirect fluorescent antibody (IFA) IgG and IgM assays with the HHV6 IgG test kit (Scimedx Corporation, Dover, NJ, USA) according to the manufacturer’s instructions. Two dilutions were used for the IgG assay, 1:40 and 1:600, as recommended by the HHV6 foundation (https://hhv-6foundation.org/patients/hhv-6-testing-for-patients, accessed on 19 March 2024). High anti-HHV6 IgG titer (in cases requiring 1:600 dilution) was determined as indicative for recent infection or reactivation [17,18]. All HHV6 RT-PCR results below CT values of 37 were considered positive.

## 3. Results

### 3.1. Incidence and Cohort Characterization

**Figure 1:**
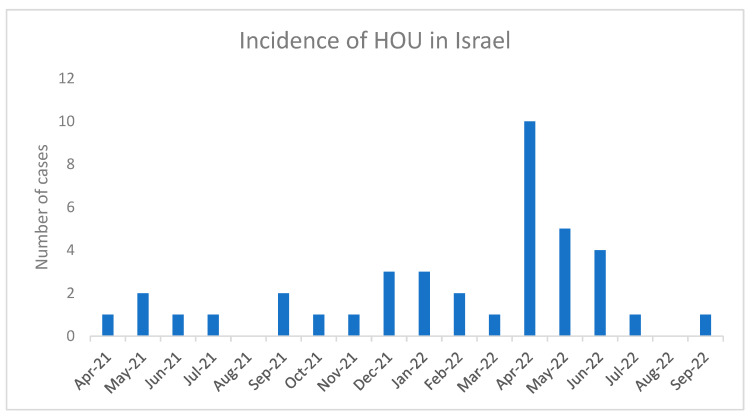
**Incidence of HUO cases in Israel.** Retrospective data were collected starting April 2021 and prospective data starting April 2022.

Upon the establishment of HUO in a national registry, pediatricians from all over the country submitted data of 62 children that presented with acute hepatitis. Four pediatric hepatologists reviewed the clinical files to exclude children who were not compatible with the inclusion criteria (e.g., liver enzymes less than 500 IU/L). Children were also excluded due to other clear diagnoses (such as autoimmune hepatitis or an established viral infection like EBV or CMV). The final cohort included a total of 39 children. The incidence of HUO cases is presented in Figure 1.

Of the 39 children, 20 (51%) were females, with a median age of 35 months. Twelve children had abdominal pain, twelve had jaundice, six were vomiting, seven had fever, and four had diarrhea. The median lab values were AST 1334 (IU/L), ALT 1240 (IU/L), total bilirubin 4.5 (mg/dL), and INR 1.14 (Table 1). Eight children were treated with steroids due to suspicion of autoimmune hepatitis and eminent liver failure. Two children underwent liver transplantation.

### 3.2. Virology and Biopsy Results

The retrospective analysis excluded common hepatitis viruses (A-E), HIV, EBV, and CMV in all these cases. Ten children underwent liver biopsy; eight of them showed variable autoimmune-mediated features consisting of plasma cells and variable degrees of interface hepatitis and two appeared as non-specific hepatitis.

Samples from 20 children were prospectively assessed by the CVL. All virological findings are summarized in Table 2. Adenovirus was found in 4 patients (4/19, 21%). One of these samples could be sequenced and was identified as human adenovirus 2 (HAdV-2). AAV2 was detected in two of sixteen patients (12.5%). These children were adenovirus negative; however, both were exposed to COVID-19.

Overall, blood samples from eleven children (58%) were HHV6 positive: six were PCR-positive, three had high anti-HHV6 IgG levels, and two were both PCR positive and had high levels of IgG. One of the patients was detected to have both HHV6 and adenovirus. Only one of the patients was negative to all tested viruses.

## 4. Discussion

Following the WHO report of the HOU outbreak, sporadic cases were reported worldwide [19], and a registration of Israeli cases was initiated. Indeed, elevation in the number of cases was previously reported [20].

Since the first report of hepatitis of unknown cause in children, many studies in different world regions were executed aiming to identify a cause and to elucidate the pathogenesis of this outbreak. However, the exact cause of HUO outbreak remains uncertain, highlighting the need for the rapid dissemination of results and stronger international collaborative efforts [21].

Different viral pathogens were shown to be linked to this outbreak, but the most stringent evidence suggested that the main suspect is adenovirus [4,5,6], with or without AAV2. In some studies, HHV6 was also reported, but only in the minority of cases. Here, we show that HHV6 was involved in many of the cases. While most children showed evidence of exposure to COVID-19, both adenovirus and AAV2 were rarely found. Adenovirus was suggested as the cause of HUO in early investigation reports from Alabama [22] and the United Kingdom [23], but until recently, adenovirus was not regarded as a cause for severe hepatitis in immune-competent children, and thus, the direct impact of this virus on current HUO was questionable. However, later studies increased the likelihood that adenovirus may play a role in the pathogenesis of HUO [4,5,6]. Servellita et al. [6] showed that in the United States, AAV2 was significantly prevalent in children with HUO, but this coincided with additional viruses such as HHV6, which were found in 50% of cases, possibly suggesting that AAV2 acts as a helper virus. Morfopoulou et.al [5] and Ho et al. [4] suggested that in the United Kingdom, AAV2 was the main virus and adenovirus acted as the helper virus, and both viruses together triggered immune-mediated inflammation against a background of HLA class II-related disease susceptibility. In these studies, HHV6 was also found, and although it was not regarded as the main cause of hepatitis, the relevance of HHV6 was not totally refuted, and HHV6 was suggested as another helper virus to AAV2.

The higher percentage of children positive for HHV6 in our study, along with the very low frequency of identifying adenovirus or AAV2 in our cohort, raises the option of HHV6 indeed having a role in the pathogenesis of HUO, at least in some world regions.

Recently, in the United Kingdom, three cases of infants (aged between 6 and 11 months) who presented with acute hepatitis and rapid progression to acute liver failure requiring liver transplantation were reported to have primary HHV6 infection [24]. The reactivation of HHV6 and acute liver failure against the background of COVID-19 has also recently been described in a four-year-old girl in Tunisia [25]. In the current cohort, most of the children were older than two years old (31/39 in total; 9/11 of the patients with HHV6), an age when most children are likely to have already been infected with the virus [7]. Therefore, it is plausible that in at least some of this cases, HHV6 is involved via its reactivation rather than primary infection and that this reactivation, against the background of the COVID-19 pandemic, resulted in this HUO. Shafiee et al. [26] recently summarized a meta-analysis showing that the cumulative incidence of HHV-6 among COVID-19 patients was 18%, suggesting possible linkage between the two pathogens; however, any relation to liver diseases was not explored. Others show that herpes viruses reactivation could occur in both acute and past COVID-19 cases [27]. Indeed, here, 7 of the children detected with HHV6 (7/11) had been exposed to COVID-19.

The involvement of the immune system in this HUO cannot be disregarded, especially as in different studies, different viral agents have been identified. Environmental factors such as viral infections and drugs are considered triggers for the development of autoimmunity [28] by forming neoantigens which activate T cells that generate inflammation [29]. Indeed, changes in T cells were also found in the blood of children with HUO and in liver biopsies [4]. In our cohort, when the first children with acute hepatitis presented, they were considered to have seronegative autoimmune hepatitis. In some of them, when hepatitis progressed along with worsening liver functions, liver biopsy was performed and showed some features of autoimmunity-interface hepatitis and increased plasma cells. Accordingly, some children were treated with steroids and all exhibited rapid declines in liver enzymes. Recently, Liu et al. [30] have shown evidence supporting the notion that the cross-reactivity of clonally expanded T cells could be one of the causes of COVID-19-related autoimmune-like hepatitis, including pediatric hepatitis of unknown etiology.

There are several limitations to our study. As the registry was established more than six months after the outbreak began, we could only prospectively analyze samples for a part of the cohort. Nevertheless, all children underwent standard investigation in the treating medical center, and possible known and common causes for acute hepatitis were excluded. The RT-PCR HHV6 assay was qualitative and did not differentiate between HHV6A and HHV6B [17], with the latter being more commonly implicated in liver diseases [5]. Indeed, the characterization of the virus in liver biopsies should be studied in future cases. Another limitation is a lack of HHV6-positive background in healthy controls. However, the reported prevalence of HHV6 in healthy children at nearly the same ages as our cohort was 37–38% by PCR in other countries [31,32]. In addition, although we appreciate the importance of HLA typing to better understand the immune background and the pathogenesis on the disease in our cohort, due to technical reasons, HLA analysis was not performed.

In conclusion, here, we report an analysis of a nationwide cohort of unexplained hepatitis in children in which HHV6 was the most abundant virus identified. Against the background of the COVID-19 pandemic and in view of various viruses reported as a plausible causes, it may be that the combination of reactivation or active infection with any virus is a trigger for the activation of the immune system, leading to acute hepatitis and this HUO outbreak.

## Figures and Tables

**Table 1:** Characterization of Israeli HUO patients.

Total	39 (%)
**Gender**	
Female	20 (51%)
Male	19 (49%)
**Age, months—mean (STD), median**	51.6 (46.2), 35
≤60	29 (74%)
>60	10 (26%)
**Laboratory results—mean (STD)**	
Aspartate aminotransferase (AST, U/L)	1516 (1193)
Alanine aminotransferase (ALT, U/L)	1455 (1006)
Gamma-glutamyl transferase (GGT, U/L)	143 (193)
Albumin (gr/dL)	3.9 (0.6)
Bilirubin total (mg/dL)	5 (5.2)
INR	2.3 (5.8)

**Table 2:** Virological results in samples from Israeli HUO patients.

Patient Number	AAV2	Adeno	COVID-19	HHV6	Any Virus	Other
**1**						
**2**						
**3**						Rhino *
**4**				Blood		
**5 ^S^**				Blood		
**6**				Blood, serum		
**7**		Feces				Rhino *
**8**				Serum		
**9**	Blood			Blood, serum		
**10**						Rhino *
**11**		Blood		Blood		Adeno *^,^**
**12 ^T^**						
**13**				Serum		
**14 ^T^**		Blood				
**15**				Blood		
**16**				Blood		
**17**				Blood		
**18 ^S^**						
**19**		Feces				Entero **
**20**	Blood			Serum		
**Total positive**	**2/16 (12.5%)**	**4/19 (21%)**	**13/19 (68.4)**	**11/19 (57.9%)**	**18/20 (90%)**	**5/12 (42%)**

Red—positive, green—negative, gray—not performed. Viruses in blood, feces, and nasopharyngeal samples were detected by PCR. In serum, high positive HHV6 IgG was determined by IFA (1:600 dilution). COVID-19 exposure was recorded according to Ministry of Health registration of positive PCR or presence of COVID-19 IgG. *—nasopharyngeal samples, **—feces samples, S—treated with steroids, T—liver transplantation.

## Data Availability

The original contributions presented in the study are included in the article and Supplementary Materials, further inquiries can be directed to the corresponding author.

## Supplementary Materials

The following supporting information can be downloaded at: https://www.mdpi.com/article/10.3390/v16050808/s1, Table S1: Samples from 20 of the patients sent to the CVL.

## References

[1] Zhaori G. (2022). Severe acute hepatitis of unknown causes in children—Current findings, questions, opinions, and recommendations, a mini-review. Pediatr. Investig..

[2] Centers for Disease Control and Prevention (2022). Children with Hep Atitis of Unknown Cause. https://www.cdc.gov/media/releases/2022/s0518-acute-hepatitis.html.

[3] Elsheikh R., Tien H.T., Makram A.M., Van N.T., Le T.T.B., Vasanthakumaran T., Huy N.T. (2023). Acute hepatitis of unknown origin in children: Behind the statistics. Hepatology.

[4] Ho A., Orton R., Tayler R., Asamaphan P., Herder V., Davis C., Tong L., Smollett K., Manali M., Allan J. (2023). Adeno-associated virus 2 infection in children with non-A-E hepatitis. Nature.

[5] Morfopoulou S., Buddle S., Torres Montaguth O.E., Atkinson L., Guerra-Assuncao J.A., Moradi Marjaneh M., Zennezini Chiozzi R., Storey N., Campos L., Hutchinson J.C. (2023). Genomic investigations of unexplained acute hepatitis in children. Nature.

[6] Servellita V., Sotomayor Gonzalez A., Lamson D.M., Foresythe A., Huh H.J., Bazinet A.L., Bergman N.H., Bull R.L., Garcia K.Y., Goodrich J.S. (2023). Adeno-associated virus type 2 in US children with acute severe hepatitis. Nature.

[7] Zerr D.M., Meier A.S., Selke S.S., Frenkel L.M., Huang M.L., Wald A., Rhoads M.P., Nguy L., Bornemann R., Morrow R.A. (2005). A population-based study of primary human herpesvirus 6 infection. N. Engl. J. Med..

[8] De Bolle L., Naesens L., De Clercq E. (2005). Update on human herpesvirus 6 biology, clinical features, and therapy. Clin. Microbiol. Rev..

[9] Phan J., Eslick G.D., Elliott E.J. (2024). Demystifying the global outbreak of severe acute hepatitis of unknown aetiology in children: A systematic review and meta-analysis. J. Infect..

[10] Hayes L.J., Uri H., Bojkova D., Cinatl J., Wass M.N., Michaelis M. (2023). Impact of the COVID-19 pandemic on the circulation of other pathogens in England. J. Med. Virol..

[11] Leshem E., Afek A., Kreiss Y. (2020). Buying Time with COVID-19 Outbreak Response, Israel. Emerg. Infect. Dis..

[12] Cooper S., Tobar A., Konen O., Orenstein N., Kropach Gilad N., Landau Y.E., Mozer-Glassberg Y., Bar-Lev M.R., Shaoul R., Shamir R. (2022). Long COVID-19 Liver Manifestation in Children. J. Pediatr. Gastroenterol. Nutr..

[13] Brosh-Nissimov T., Benshalom-Tirosh N., Bucris E., Morad H., Zuckerman N.S., Tepperberg Oikawa M. (2022). Recurrent congenital cytomegalovirus infection in a sequential pregnancy with severe sequelae, and a possible association with prophylactic valacyclovir treatment: A case report. Int. J. Infect. Dis..

[14] Erster O., Bar-Or I., Levy V., Shatzman-Steuerman R., Sofer D., Weiss L., Vasserman R., Fratty I.S., Kestin K., Elul M. (2022). Monitoring of Enterovirus D68 Outbreak in Israel by a Parallel Clinical and Wastewater Based Surveillance. Viruses.

[15] Heim A., Ebnet C., Harste G., Pring-Akerblom P. (2003). Rapid and quantitative detection of human adenovirus DNA by real-time PCR. J. Med. Virol..

[16] Aurnhammer C., Haase M., Muether N., Hausl M., Rauschhuber C., Huber I., Nitschko H., Busch U., Sing A., Ehrhardt A. (2012). Universal real-time PCR for the detection and quantification of adeno-associated virus serotype 2-derived inverted terminal repeat sequences. Hum. Gene Ther. Methods.

[17] Foundation, HHV-6. Overview on Testing for HHV-6 Infection. https://hhv-6foundation.org/patients/hhv-6-testing-for-patients.

[18] Yamane A., Mori T., Suzuki S., Mihara A., Yamazaki R., Aisa Y., Nakazato T., Shimizu T., Ikeda Y., Okamoto S. (2007). Risk factors for developing human herpesvirus 6 (HHV-6) reactivation after allogeneic hematopoietic stem cell transplantation and its association with central nervous system disorders. Biol. Blood Marrow Transplant..

[19] Matthews P.C., Campbell C., Sandulescu O., Maticic M., Ruta S.M., Rivero-Juarez A., van Welzen B.J., Tan B.K., Garcia F., Gherlan G.S. (2022). Acute severe hepatitis outbreak in children: A perfect storm. What do we know, and what questions remain?. Front. Pharmacol..

[20] van Beek J., Fraaij P., Giaquinto C., Shingadia D., Horby P., Indolfi G., Koopmans M. (2022). Case numbers of acute hepatitis of unknown aetiology among children in 24 countries up to 18 April 2022 compared to the previous 5 years. Euro Surveill.

[21] Lewis L., van Wylick C., Mulder D.J. (2023). A systematic review of the proposed etiologies of the 2021–2022 outbreaks of pediatric acute hepatitis of unknown etiology. Front. Pediatr.

[22] Baker J.M., Buchfellner M., Britt W., Sanchez V., Potter J.L., Ingram L.A., Shiau H., Sanchez L.H.G., Saaybi S., Kelly D. (2022). Acute hepatitis and adenovirus infection among children-Alabama, October 2021-February 2022. Am. J. Transpl..

[23] Kelgeri C., Couper M., Gupte G.L., Brant A., Patel M., Johansen L., Valamparampil J., Ong E., Hartog H., Perera M. (2022). Clinical Spectrum of Children with Acute Hepatitis of Unknown Cause. N. Engl. J. Med..

[24] Warner S., Brown R.M., Reynolds G.M., Stamataki Z., Kelly D.A. (2023). Case report: Acute liver failure in children and the human herpes virus 6-? A factor in the recent epidemic. Front. Pediatr..

[25] Borgi A., Ayari A., Hajji A., Louati A., Bouziri A., Menif K., Jaballah N.B. (2021). Reactivation of Human Herpes Virus 6 and Acute Liver Failure in Multisystem Inflammatory Syndrome. Indian J. Pediatr..

[26] Shafiee A., Teymouri Athar M.M., Amini M.J., Hajishah H., Siahvoshi S., Jalali M., Jahanbakhshi B., Mozhgani S.H. (2023). Reactivation of herpesviruses during COVID-19: A systematic review and meta-analysis. Rev. Med. Virol..

[27] Banko A., Miljanovic D., Cirkovic A. (2023). Systematic review with meta-analysis of active herpesvirus infections in patients with COVID-19: Old players on the new field. Int. J. Infect. Dis..

[28] Floreani A., Restrepo-Jimenez P., Secchi M.F., De Martin S., Leung P.S.C., Krawitt E., Bowlus C.L., Gershwin M.E., Anaya J.M. (2018). Etiopathogenesis of autoimmune hepatitis. J. Autoimmun..

[29] Fujinami R.S., von Herrath M.G., Christen U., Whitton J.L. (2006). Molecular mimicry, bystander activation, or viral persistence: Infections and autoimmune disease. Clin. Microbiol. Rev..

[30] Liu Y., Wang Y., Peng Z., Li G., Wang J. (2023). T cell cross-reactivity in autoimmune-like hepatitis triggered by COVID-19. hLife.

[31] Dossier C., Sellier-Leclerc A.L., Rousseau A., Michel Y., Gautheret-Dejean A., Englender M., Madhi F., Charbit M., Ulinski T., Simon T. (2014). Prevalence of herpesviruses at onset of idiopathic nephrotic syndrome. Pediatr. Nephrol..

[32] Inoue J., Weber D., Fernandes J.F., Adegnika A.A., Agnandji S.T., Lell B., Kremsner P.G., Grobusch M.P., Mordmuller B., Held J. (2023). HHV-6 infections in hospitalized young children of Gabon. Infection.

