# Supplementary material for: The Outbreak of Unexplained Acute Hepatitis in Children: The Role of Viral Infections in View of the COVID-19 Pandemic
Source: Viruses. 2024 May 20;16(5):808. doi: 10.3390/v16050808 (PMC11125843; doi:10.3390/v16050808)
Supplement: Supplementary file 1 [file viruses-16-00808-s001.zip › viruses-2952878-supplementary.pdf]

Table S1: Samples from 20 of the patients sent to the CVL

Patient number	Blood	Serum	Nasopharyngeal swab	Feces
1	X	X	X	
2		X		X
3	X	X	X	X
4	X	X		
5	X	X	X	
6	X	X	X	X
7	X	X	X	X
8	X	X		
9	X	X		
10	X		X	
11	X	X	X	
12	X			
13	X	X	X	X
14	X	X		
15	X	X	X	X
16	X	X	X	
17	X	X	X	X
18	X			
19	X		X	X
20	X	X	X	
